# Fundamental concerns of women living with HIV around the implementation of Option B+

**DOI:** 10.7448/IAS.18.6.20286

**Published:** 2015-12-01

**Authors:** Rebecca Matheson, Suzette Moses-Burton, Amy C Hsieh, Sophie Dilmitis, Margaret Happy, Eunice Sinyemu, Sophie O Brion, Aditi Sharma

**Affiliations:** 1 International Community of Women Living with HIV (ICW), Nairobi, Kenya; 2 Global Network of People Living with HIV (GNP+), Amsterdam, The Netherlands; 3 Global Network of People Living with HIV (GNP+), Brooklyn, NY, USA; 4 International Community of Women Living with HIV (ICW), Harare, Zimbabwe; 5 International Community of Women Living with HIV (ICW), Kampala, Uganda; 6 International Community of Women Living with HIV (ICW), Lusaka, Zambia; 7 Network of Zambian People Living with HIV (NZP+), Lusaka, Zambia; 8 International Community of Women Living with HIV (ICW), Washington, DC, USA; 9 Global Network of People Living with HIV (GNP+), Brighton, UK

**Keywords:** adherence, eMTCT, implementation science, PMTCT, qualitative research, retention, vertical transmission

## Abstract

**Introduction:**

In 2011, the Global Plan towards the Elimination of New HIV Infections among Children by 2015 and Keeping Their Mothers Alive was launched to scale up efforts to comprehensively end vertical HIV transmission and support mothers living with HIV in remaining healthy. Amidst excitement around using treatment as prevention, Malawi's Ministry of Health conceived Option B+, a strategy used to prevent vertical transmission by initiating all pregnant and breastfeeding women living with HIV on lifelong antiretroviral therapy, irrespective of CD4 count. In 2013, for programmatic and operational reasons, the WHO officially recommended Option B+ to countries with generalized epidemics, limited access to CD4 testing, limited partner testing, long breastfeeding duration or high fertility rates.

**Discussion:**

While acknowledging the opportunity to increase treatment access globally and its potential, this commentary reviews the concerns of women living with HIV about human rights, community-based support and other barriers to service uptake and retention in the Option B+ context. Option B+ intensifies many of the pre-existing challenges of HIV prevention and treatment programmes. As women seek comprehensive services to prevent vertical transmission, they can experience various human rights violations, including lack of informed consent, involuntary or coercive HIV testing, limited treatment options, termination of pregnancy or coerced sterilization and pressure to start treatment. Yet, peer and community support strategies can promote treatment readiness, uptake, adherence and lifelong retention in care; reduce stigma and discrimination; and mitigate potential violence stemming from HIV disclosure. Ensuring available and accessible quality care, offering food support and improving linkages to care could increase service uptake and retention. With the heightened focus on interventions to reach pregnant and breastfeeding women living with HIV, a parallel increase in vigilance to secure their health and rights is critical.

**Conclusion:**

The authors conclude that real progress towards reducing vertical transmission and achieving viral load suppression can only be made by upholding the human rights of women living with HIV, investing in community-based responses, and ensuring universal access to quality healthcare. Only then will the opportunity of accessing lifelong treatment result in improving the health, dignity and lives of women living with HIV, their children and families.

## Introduction

Option B+ could be good, but we still lack information. – Woman living with HIV, Uganda [1]

In 2011, the Global Plan towards the Elimination of New HIV Infections among Children by 2015 and Keeping Their Mothers Alive (Global Plan) was launched to scale up efforts to comprehensively end vertical HIV transmission and support mothers living with HIV in remaining healthy [2]. This undertaking re-emphasized the necessity of the four-pillared approach, also known as *prevention-of-mother-to-child-transmission* [3, 4], (The term *comprehensive prevention of vertical transmission* is used where possible in this commentary in line with developing usage among the community of people living with HIV. For a fuller discussion on terminology, see Refs. [3, 4]), which aims to 1) prevent HIV acquisition among women of reproductive age; 2) provide appropriate care to meet the family planning needs of women living with HIV; 3) support pregnant women living with HIV to prevent vertical transmission; and 4) provide care, treatment and support to women living with HIV, their children and families [5].

Concurrently, excitement around treatment as prevention was mounting [6–8]. Against this backdrop in 2011, Malawi's Ministry of Health conceived and began implementing a new strategy to prevent vertical transmission that provides lifelong antiretroviral therapy (ART) to all pregnant and breastfeeding women living with HIV, irrespective of their CD4 count. Expanding on an existing WHO recommendation to HIV programme managers called *Option B*, which offers triple combination antiretrovirals (ARVs) as prophylaxis only during pregnancy and breastfeeding, Malawi's approach was dubbed *Option B+*. Malawi's primary rationale was the lack of access to CD4 cell count equipment and systems for successful referral to ART services for women who need treatment for their own health, the risk of viral rebound after ARV cessation and inconsistent breastfeeding cessation patterns, together causing increased HIV acquisition risk to a sexual partner or child [9, 10].

In 2013, the WHO officially recommended Option B+, for programmatic and operational reasons, particularly in countries with generalized epidemics, high fertility rates or long breastfeeding duration [11]. These were “conditional recommendations” based on “low-quality evidence” [11]. WHO additionally reasoned that Option B+ offered simplified implementation with a standardized drug regimen (the same as for non-pregnant individuals) and was cost-effective [11].

In November 2014, world leaders launched the Fast-Track Strategy to End the AIDS Epidemic by 2030, in which Option B+ plays a leading role [12]. To date, 19 of 22 Global Plan countries have committed to Option B+, though most are in the early or scale-up phases of implementation [13].

Communities of people living with HIV [1, 14–16] and clinicians [17–19] have held mixed and evolving perceptions of Option B+ since its inception. Women living with HIV understand the opportunity Option B+ presents to increase treatment access globally and its potential (e.g. improved mothers’ and babies’ health, reduced stigma, opportunities to breastfeed). However, few articles discuss the need to uphold human rights, invest in community-based support and address barriers to service uptake and retention as being essential in order to comprehensively end vertical transmission. This commentary reviews these concerns raised by women living with HIV regarding Option B+ implementation and calls for funding to support its examination through women's lived experiences.

## Discussion

As global momentum and pressure on countries to meet Global Plan targets have increased, so has the pressure on pregnant and breastfeeding women to be tested for HIV and initiate early and lifelong treatment. Option B+ intensifies many of the pre-existing challenges of HIV prevention and treatment programmes. With the heightened focus on interventions to reach pregnant and breastfeeding women, a parallel increase in vigilance to secure their health and rights is critical.

### Upholding human rights

Before I start on B+, I should be informed of all options and all of the advantages … so that I make informed decisions based on the benefits that are there. – Woman living with HIV, Uganda [1]

A primary concern regarding Option B+ implementation is whether women's human rights are upheld and what the consequences are for rights violations. A rights-based approach to end vertical transmission requires, at a minimum, that services be consistent with international human rights obligations [20–27]. Accordingly, women living with HIV have an autonomous right to make fully informed and voluntary decisions about whether to have an HIV test, learn the result, start or opt out of treatment, receive support for whichever decision they make and determine their sexual and reproductive lives free from stigma and discrimination [28–32]. During a 2012 consultation held in Malawi and Uganda, women living with HIV expressed concern about whether women are offered sufficient information about why and how to start lifelong treatment, time to reflect and the opportunity to choose [1].

A South Africa programme is pilot testing a “rapid ART initiation approach” as part of Option B+ [33]. In this method, women are enrolled in ART within the same week of their first antenatal care visit [33, 34]. The programme boasts a 97.0% ART initiation rate, with 90.8% initiating within the same day that treatment eligibility is determined [33].

However, given insufficient time to process the news, along with the information asymmetry and power disparity between client and provider [35], women in Malawi have reported feeling pressured to accept lifelong treatment without being fully informed of possible side effects, understanding the commitment and having linkage to care and support for adherence [1]. Such rapid initiation of lifelong treatment requires analysis as to whether the process meets international human rights standards for informed consent.

Across the four pillars, women can experience a spectrum of coercive practices, including lack of informed consent, involuntary or coercive HIV testing [36, 37], limited contraception [38] or treatment options, termination of pregnancy or coerced sterilization [39–44] and pressure to accept particular contraceptives or start treatment [37, 45]. These practices lead to disempowerment around testing and treatment choices, which can discourage women from seeking care and are counterproductive to meeting public health goals [30].

Indeed, early loss to follow-up has been a challenge for many countries implementing Option B+. For instance, in Malawi, compared to individuals who started ART for their own health, women who started ART while pregnant were five times less likely to return to the clinics after the initial visit (when they initiated ART) [46]. On average, 17 and 22% of all pregnant women starting ART under Option B+ dropped out of care in the first six months and year of therapy, respectively [46]. Women who started ART while breastfeeding were twice as likely to miss their first follow-up visit [46].

Additionally, non-discriminatory care, free from HIV-related stigma, is also vital to comprehensively prevent vertical transmission. Unlike previous prophylactic protocols, women must feel comfortable and supported to begin treatment “earlier” and remain in lifelong care. Yet, women reported receiving discriminatory care, where service providers do not treat them with dignity (e.g. yelling derogatory statements) and violate other human rights (e.g. disclosing serostatus, failing to provide correct or full information) [47]. Such discriminatory care provided by clinic staff to clients during labour and delivery and when they collected ARVs preceded the rollout of Option B+ in Malawi and remains a primary concern for women living with HIV [48].

Additionally, women are concerned that Option B+ may exacerbate inequities around treatment access by men and non-pregnant women [1, 49]. Some women in Malawi and Uganda feared the potential increase in domestic violence if one partner discovers her serostatus and/or starts treatment before the other and is accused of “bringing the virus home” [1]. Yet, policies that have attempted to engage partners (e.g. Uganda's requirement for pregnant women to bring their partners to antenatal visits) have also produced unintended consequences. As one Ugandan woman shared, “Women have chose[n] to hire *boda boda* (motorcycle taxi drivers) to go with them to access the services” [1]. Consequently, countries implementing Option B+ must consider how to prevent negative consequences of ART prioritization and design partner engagement policies that do not put women at greater risk.

Improving the circumstances around offering lifelong ART, by focusing on quality rather than quantity, is needed to support women to make informed decisions regarding timely treatment initiation and to remain in lifelong care [50]. In particular, programmes seeking to end vertical transmission should 1) involve women living with HIV in programme design and implementation; 2) train healthcare workers to provide non-discriminatory care, provide sufficient information and obtain informed consent without coercion; and 3) provide mechanisms for women to raise and address concerns about human rights violations (e.g. patient representatives, healthcare facility ombudsman or complaints mechanism). Finally, funding networks of women living with HIV, to provide needed information, rights education and other support, provides an additional opportunity to ensure that human rights are respected, protected and fulfilled.

### Investing in community-based support

There should be education for everybody that states clearly when people should start treatment so people are prepared. The doctors and those of us in support groups know a little but we need to disseminate the information. – Woman living with HIV, Malawi [1]

Community-based support is critical to Option B+ implementation. Communities play an important role in facilitating treatment readiness, support and retention in care. After years of being told to wait before starting ART, women need education and awareness of the benefits of earlier treatment initiation for their own health and how to manage lifelong treatment and side effects.

Personal readiness to start treatment is complex and motivated by many personal and social factors [51, 52]. Women living with HIV from Malawi and Uganda have warned that starting patients on treatment before they feel ready would not be conducive to adherence, retention or good health [1]. In Tanzania, women acknowledged the eventual need for treatment for their own health, but shared that they may lose motivation to remain in treatment after the risk of transmission to the child has passed, due to fear of the medications’ side effects and not feeling ready to remain on lifelong treatment [53]. Additionally, studies have suggested that more women who start ART for their own health remain in care than those who start for other reasons [54, 55]. Yet, as with treatment programmes that existed before Option B+, stigma and discrimination at home and in the community, divorce and physical violence have caused many women to decline treatment or hide their medications to prevent unintended disclosure [56, 57].

Peer and community support strategies can promote treatment uptake, adherence and lifelong retention in care [1, 46, 58–64]. Peer-to-peer strategies can further help to reduce stigma and discrimination and mitigate potential violence stemming from disclosure of HIV status [1]. High quality education and support groups meeting at the hospital or in the villages have been found to facilitate access and retention in care [48]. Community-based follow-up, such as home visits with women–infant pairs as in Zambia [65], has also improved antenatal care attendance. Women from Malawi and Uganda highlighted the need for clear information and education in communities, peer-to-peer counselling and community-led retention and adherence models to improve literacy, preparedness [51] and agency in order to enable women to assert their rights [1].

### Overcoming barriers to service uptake and retention

If I am coming from some place and you are referring me 40 kilometers, you find that the mother does not have that transport and this will hurt adherence. – Woman living with HIV, Uganda [1]

Women who commence lifelong ART, especially those with young children, shoulder new burdens arising from long-term routine clinic appointments. With Option B+, women experience temporal, financial (i.e. transportation costs, missing work), relational (e.g. permission from partner), emotional and physical (e.g. side effects) costs for the remainder of their lives. These costs affect women's ability to seek HIV care and adhere to treatment regimens [1] and have caused some women in Malawi to stop ART [66].

When women have been able to attend appointments, dysfunctional clinic and health systems – including healthcare worker shortages, long wait times and distances to clinics – disincentivize them from seeking care [67]. Studies in Tanzania and Malawi highlighted that maintenance of regular supplies of HIV-related test kits and medications are important in ensuring that all women in need of vertical transmission prevention services are reached [35, 67, 68].

Women living with HIV affirmed that ensuring available and easily accessible quality care, particularly by having follow-up and ARV distribution points closer to communities, could increase service uptake [1]. Access to food and food support (e.g. supplements for their infants, money from support groups) are also important facilitators to care [48]. A study of 141 health facilities in Malawi found that the number of women per HIV testing and counseling (HTC) counsellor, HIV-related test kit availability and the “model of care” affected treatment uptake; district location, patient volume and the model of care affected retention in care [69]. Improving linkages between antenatal and ARV services is important [70–73], but further research with women living with HIV is crucial to determining which models of care will most successfully support treatment initiation and lifelong ART [69].

## Conclusions

Option B+ has expanded treatment access for many pregnant and breastfeeding women living with HIV. However, real progress towards reducing vertical transmission and achieving viral load suppression can only be made by upholding the human rights of women living with HIV, investing in community-based responses and ensuring universal access to quality healthcare. Only then will the opportunity of accessing lifelong treatment result in improving the health, dignity and lives of women living with HIV, their children and families.

To meet Global Plan targets, governments and programmes seeking to end vertical transmission must do the following:

*Uphold human rights* by ensuring that service providers 1) always provide women sufficient information and time to make informed decisions regarding treatment for their health and the health of the child, 2) do not coerce women into accepting lifelong treatment should another option be desired, 3) respect confidentiality and 4) provide non-discriminatory care.

*Invest in community-based responses* to improve linkages to services, treatment literacy, preparedness and agency to enable women to receive quality services and adhere to treatment. This investment includes financial support to deliver community-based services, which are the backbone of healthcare systems, support groups, peer supporters and mentor mothers living with HIV, and linkages to networks and organizations of women living with HIV.

*Overcome barriers to service uptake and retention* by ensuring access to quality healthcare and providing decentralized services to address common challenges (e.g. distance to clinics, transport costs and long waiting times). This includes ensuring adequate supplies of ARVs, other medications and diagnostic tools as well as integration with other programmes (e.g. maternal and child health, nutrition, mental health) in ways that deliver the best care for women living with HIV and their families.

The experience and meaningful involvement of women living with HIV regarding design, implementation, monitoring and evaluation of Option B+ are crucial to comprehensively prevent vertical transmission. Teresia Njoki Otieno, speaking as ICW Global Chair at the launch of the Fast-Track strategy, reaffirmed, “We should end this epidemic, but we can only do this if we put women living with HIV at the centre”.
